# Metabolic Evaluation of the Dietary Guidelines’ Ounce Equivalents of Protein Food Sources in Young Adults: A Randomized Controlled Trial

**DOI:** 10.1093/jn/nxaa401

**Published:** 2021-03-09

**Authors:** Sanghee Park, David D Church, Scott E Schutzler, Gohar Azhar, Il-Young Kim, Arny A Ferrando, Robert R Wolfe

**Affiliations:** Department of Geriatrics, Donald W. Reynolds Institute on Aging, University of Arkansas for Medical Sciences, Little Rock, AR, USA; Department of Molecular Medicine, Lee Gil Ya Cancer and Diabetes Institute, College of Medicine, Gachon University, Incheon, South Korea; Department of Geriatrics, Donald W. Reynolds Institute on Aging, University of Arkansas for Medical Sciences, Little Rock, AR, USA; Department of Geriatrics, Donald W. Reynolds Institute on Aging, University of Arkansas for Medical Sciences, Little Rock, AR, USA; Department of Geriatrics, Donald W. Reynolds Institute on Aging, University of Arkansas for Medical Sciences, Little Rock, AR, USA; Department of Molecular Medicine, Lee Gil Ya Cancer and Diabetes Institute, College of Medicine, Gachon University, Incheon, South Korea; Department of Geriatrics, Donald W. Reynolds Institute on Aging, University of Arkansas for Medical Sciences, Little Rock, AR, USA; Department of Geriatrics, Donald W. Reynolds Institute on Aging, University of Arkansas for Medical Sciences, Little Rock, AR, USA

**Keywords:** ounce equivalent, anabolic response, essential amino acids, net protein balance, stable isotope tracers

## Abstract

**Background:**

The Dietary Guidelines for Americans (DGAs) published an “ounce equivalents” recommendation to help consumers meet protein requirements with a variety of protein food sources. However, the metabolic equivalency of these varied protein food sources has not been established.

**Objective:**

We have investigated the hypothesis that the anabolic responses to consumption of ounce equivalents of protein food sources would be directly related to the essential amino acid (EAA) content of the protein food source.

**Methods:**

Following 3 d of dietary control, a total of 56 healthy young adults underwent an 8.5-h metabolic study using stable isotope tracer methodology. The changes from baseline following consumption of 1 of 7 different protein food sources were compared with the baseline value for that individual (*n* = 8 per group).

**Results:**

Consumption of ounce equivalents of animal-based protein food sources (beef sirloin, pork loin, eggs) resulted in a greater gain in whole-body net protein balance above baseline than the ounce equivalents of plant-based protein food sources (tofu, kidney beans, peanut butter, mixed nuts; *P* < 0.01). The improvement in whole-body net protein balance was due to an increase in protein synthesis (*P *< 0.05) with all the animal protein sources, whereas the egg and pork groups also suppressed protein breakdown compared with the plant protein sources (*P* < 0.01). The magnitude of the whole-body net balance (anabolic) response was correlated with the EAA content of the protein food source (*P *< 0.001).

**Conclusion:**

The “ounce equivalents” of protein food sources as expressed in the DGAs are not metabolically equivalent in young healthy individuals. The magnitude of anabolic response to dietary proteins should be considered as the DGAs develop approaches to establish healthy eating patterns.

See corresponding article on page 1055.

## Introduction

Over the past 35 y, the USDA Dietary Guidelines for Americans (DGAs) have sought to translate recommendations on nutrient requirements (i.e., RDAs) from the Food and Nutrition Board of the Institute of Medicine into practical nutritional advice for the American public (1). The DGAs are intended to incorporate nutrient RDAs into complete dietary patterns. Despite the well-established importance of dietary protein, the DGAs do not place emphasis on protein nutrition. This position may reflect the fact that the RDA for dietary protein can be met with almost any Western diet that meets caloric requirements. However, the RDA expresses the minimal amount of dietary protein consumption necessary to avoid deficiencies in young, healthy individuals, and the optimal amount of dietary protein may be greater than the RDA in a variety of circumstances, including exercise training (2), aging (3), and recovery from serious illness (4). Furthermore, the DGAs do not currently address the issue of protein quality, despite the long-standing recognition of its importance. The digestible indispensable amino acid score (DIAAS) was published in 2013 by the Food and Agriculture Organization of the World Health Organization as a method to quantify protein quality (5). The DIAAS extended the principles of the protein digestibility–corrected amino acid score (PDCAAS), published by the same organization in 1990 (6). The DIAAS of a protein is determined by its true ileal digestibility and the content and profile of essential amino acids (EAAs) in relation to dietary requirements (5). In general, animal proteins have higher DIAAS than plant proteins, often by as much as 2-fold (7). This measure of protein quality indicates that animal proteins can more readily provide the daily requirement of EAAs than plant proteins (7).

MyPlate is designed to simplify for the public the key elements of the DGAs (8). MyPlate recommends meeting protein needs by eating a variety of protein food sources. To assist the consumer in meeting protein needs with varying sources of dietary protein, the DGAs present a variety of “ounce equivalents” in the protein foods group (8). The DGAs state that 1 ounce (28 g) of meat is equivalent to 1 cooked egg, ¼ cup (70 g) of red kidney beans, 1 tablespoon (15 g) of peanut butter, 2 ounces (56 g) of tofu, and 0.5 ounces (14 g) of mixed nuts. The labeling of these disparate protein food sources as “equivalents” implies an equal metabolic benefit should be obtained from each of the “ounce equivalents” of protein food sources. The basis for considering these protein food sources to be “equivalent” is unclear. Upon inspection, these “equivalent” protein food sources do not appear to be equivalent in any respect. The ounce equivalents differ in macronutrient composition, particularly in protein and EAA content, as well as in caloric value. In the current study, we used stable isotope tracer methodology to investigate the hypothesis that the anabolic response, defined as the increase in net protein balance (i.e., whole-body protein synthesis minus breakdown) differs in response to consumption of so-called ounce equivalents of protein food sources. We further have investigated the hypothesis that the gain in net body protein after consumption of various “ounce equivalents” of protein food sources is directly related to the EAA content.

## Materials and Methods

### Participants

A group size of 8 per group was a priori determined based upon an ANCOVA model to compare the protein sources with respect to mean response (anabolic response or net protein synthesis) after adjusting for baseline measures. With this sample size, the ANCOVA model had 80% power to detect effect sizes of *f* = 0.484 or larger. This estimate assumed that the baseline covariate explains 50% of the variation in the response. A 5% α level was used to determine statistical significance.

A flowchart of study participants is described in **Supplemental Figure 1**. The study was initiated on 5 May 2017 and completed on 3 June 2019. A total of 64 individuals were recruited from the Little Rock, Arkansas, area. Of those recruited, 56 participants were randomly assigned into each treatment (8 per group); 5 participants were excluded due to failure to meet inclusion/exclusion criteria, and 3 dropped out before any data were collected.

Participant eligibility for the study was based on a battery of medical tests, including medical history, blood count, and medical questionnaires. Participants who were aged 18 to 40 y with a BMI (in kg/m^2^) of 20 to 30 were included. Exclusion criteria included participants with current pregnancy, unwillingness to eat animal proteins, a food allergy to any of the test proteins, current use of protein or amino acid supplements, diabetes, active malignancy within the past 6 mo, gastrointestinal bypass surgery, a chronic inflammatory disease, low hemoglobin concentration, low platelets, concomitant use of corticosteroids, or any unstable medical condition. In addition, participants who performed regular exercise more than once a week were excluded. Written informed consent was obtained from all participants, and the study was approved by the Institutional Review Board at the University of Arkansas for Medical Sciences (UAMS). Participants were compensated to cover travel expenses.

### Experimental design

During the screening visit, DXA (QDR-4500A; Hologic) was performed to determine body composition in order to express whole-body protein kinetics relative to lean body mass. Participants were assigned by a study coordinator to 1 of 7 food intervention groups via a single-blinded permuted block randomization, stratified for sex: 2 ounces (56 g) of cooked beef sirloin, 2 ounces (56 g) of cooked pork loin, 2 cooked eggs, ½ cup (140 g) of red kidney beans, 2 tablespoons (30 g) of peanut butter, 4 ounces (112 g) of tofu, and 1 ounce (28 g) of mixed nuts. The 2 meats were cooked to temperature in a skillet and then portioned out. The kidney beans came precooked. Twice the amount of each ounce equivalent was used to ensure a measurable response. Participant characteristics are shown in **Table 1**, and macronutrient data for all the protein food sources are shown in **Table 2**.

**TABLE 1:** Participant demographics for each intervention group^1^

Characteristic	Beef Sirloin	Eggs	Pork Loin	Kidney Beans	Peanut Butter	Tofu	Mixed Nuts
Participant number (M/F)	8 (4/4)	8 (4/4)	8 (4/4)	8 (4/4)	8 (4/4)	8 (4/4)	8 (4/4)
Age, y	23.9 ± 1.6	23.9 ± 1.9	22.1 ± 1.0	23.8 ± 1.9	20.3 ± 1.5	25.9 ± 2.2	24.3 ± 2.1
Body weight, kg	68.0 ± 4.0	74.2 ± 5.4	74.3 ± 3.5	69.8 ± 5.7	70.4 ± 3.8	75.9 ± 5.2	74.3 ± 5.3
BMI, kg/m^2^	23.5 ± 1.0	24.4 ± 1.3	24.5 ± 0.9	24.1 ± 1.7	24.1 ± 1.1	25.9 ± 1.0	24.9 ± 1.2
Lean body mass, kg	43.5 ± 3.3	49.1 ± 3.4	51.8 ± 4.6	44.8 ± 3.1	48.1 ± 3.8	49.7 ± 4.2	49.3 ± 3.9
Fat mass, %	31.0 ± 2.4	27.4 ± 2.5	29.9 ± 3.9	30.0 ± 2.5	27.1 ± 2.3	33.0 ± 3.0	32.3 ± 2.2

^1^Values are expressed as means ± SEMs, *n* = 8 per group.

**TABLE 2:** Macronutrient composition of two “ounce-equivalent” protein food sources

Characteristic	Beef Sirloin	Eggs	Pork Loin	Tofu	Kidney Beans	Peanut Butter	Mixed Nuts
Calories, kcal	103.8	155.0	118.5	79.1	112.4	188.2	168.7
Protein, g	17.3	12.6	16.2	9.3	7.7	8.0	4.9
% of total macronutrients^1^	76	52	75	58	27	26	18
Carbohydrate, g	0.0	1.1	0.0	1.9	20.2	7.0	7.2
% of total macronutrients^1^	0	5	0	12	71	23	27
Fat, g	5.5	10.6	5.5	4.7	0.4	16.0	14.6
% of total macronutrients^1^	24	44	25	30	1	52	55
Total EAA^2^	7.0	5.6	7.1	3.8	3.0	2.0	1.8
% of total protein	40	44	44	41	39	25	37
Protein food mass, g	56.7	100.	56.7	113.	88.5	32.0	28.4

1 Expressed as percent of the sum of protein, carbohydrate, and fat calories.

2 Essential amino acids. Values are from the USDA Nutrient Data Base.

Participants consumed a weight maintenance diet for 3 d prior to the study day that was prepared and packaged by a research dietitian in the metabolic kitchen at the Donald W. Reynolds Institute on Aging (RIOA) at UAMS and taken home by the participants. Participants were instructed to abstain from strenuous physical activity, such as resistance exercise or aerobic training, throughout that time period as well. Caloric intake for the diet stabilization phase was based on the Harris-Benedict equation (9) for each participant multiplied by an activity factor of 1.4, and the meals contained 1.2 g protein/(kilograms of body weight × day). Participants were given a digital camera to photograph the meal before and after consumption to ascertain study compliance. Participants returned the camera on the morning of the fourth day when they reported for the metabolic study. All participants met the compliance requirements (consumption of >80% of the meals provided).

### Stable isotope tracer infusion protocol

**FIGURE 1:**
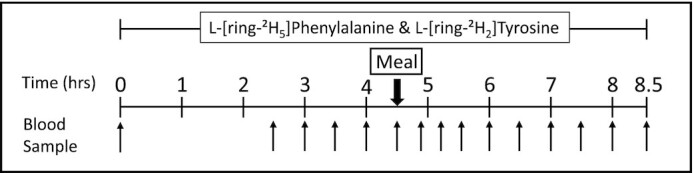
Experimental protocol.

Participants reported to the RIOA at 07:00 after an overnight fast from 22:00 the previous night and participated in a 2-period tracer infusion-metabolic study using stable isotope tracers (**Figure 1**): 4.5 h for the baseline fasted period and 4 h for the postmeal period (total 8.5-h period). Upon arrival, catheters were inserted into a vein in the forearm of one arm for tracer infusion and in a hand or wrist vein of the contralateral arm for blood sampling using the heated hand technique. After obtaining a blood sample to determine background enrichments, priming doses of L-[ring-^2^H_5_]phenylalanine, L-[ring^2^H_2_]tyrosine, and L-[ring-^2^H_4_]tyrosine were given. Infusion of L-[ring-^2^H_5_]phenylalanine and L-[ring-^2^H_2_]tyrosine was then started and maintained throughout the metabolic study. Baseline blood samples were taken before the start of the tracer infusion. Postabsorptive samples were obtained at 150, 180, 210, 240, and 270 min. Immediately after the final fasted blood sample was drawn, participants consumed 1 of the 7 protein food sources as described above. Blood samples were drawn at 290, 310, 330, 360, 390, 420, 450, 480, and 510 min of tracer infusion (postingestion blood samples) to measure tracer enrichment and plasma responses of EAAs and insulin. The tracer infusion and blood sampling protocol were identical for all participants.

### Calculation of protein kinetics

The calculation of whole-body protein kinetics (protein synthesis, protein breakdown, and net protein balance) was based on the determinations of the rate of appearance (Ra) of phenylalanine (Phe) and of tyrosine into plasma and the fractional Ra of endogenous tyrosine resulting from phenylalanine hydroxylation. A 2-pool model has been previously described and discussed in detail (10, 11). Briefly, an isotopic steady state was established in the baseline/fasted period, and protein kinetics were calculated accordingly (12). For the 4 h after ingestion of the ounce equivalent, the AUC of plasma enrichments of phenylalanine and tyrosine tracers were calculated using GraphPad Prism 6 (GraphPad Software). Ra of Phe reflects protein breakdown in the fasted state; the total appearance of Phe over the 4-h postprandial state reflects both protein breakdown and the appearance of protein from the ingested meal. The appearance of exogenous Phe in the peripheral circulation must be subtracted from total appearance of Phe to determine the rate of endogenous protein breakdown. The total appearance of exogenous Phe in peripheral blood was estimated from the amount of Phe in the dietary protein and the amount of protein consumed, the published value for the true ileal digestibility of Phe in the test protein, and the measured fraction of absorbed Phe hydroxylated to tyrosine (11). We have recently discussed in detail the validity of assumptions underlying this model of protein kinetics (11). Most important, this model does not introduce any systematic errors that might affect the ranking of the anabolic responses of the various protein food sources evaluated (11). The specific calculations have been described in detail in our previous calculations (10, 11).

### Analytic methods

Plasma samples were processed as previously described for determination of enrichment by GC-MS (models 7890A/5975; Agilent Technologies) (10). Plasma amino acid concentrations were determined by LC-MS (QTrap 5500 MS; AB Sciex) using the internal standard method as described previously (10). Plasma insulin concentrations were measured by a commercially available human insulin ELISA kit (Alpco Diagnostics).

### Statistical analysis

The postprandial change in net protein balance relative to the baseline in each group was the primary outcome. Responses of the rates of protein synthesis and breakdown were secondary outcomes. Additional secondary end points were plasma EAA and insulin AUC above baseline values, as well as protein kinetics normalized by caloric and EAA intake. Correlation analysis of the relation between protein kinetics and the EAA content of the protein food source were additional secondary end points.

One-factor repeated-measures ANOVA was used to compare differences in protein kinetics [net balance (NB), protein synthesis, and protein breakdown], plasma EAAs, insulin AUC above fasting values, and protein kinetics normalized by caloric and EAA intake. All significant main effects of groups were followed with Bonferroni-adjusted pairwise comparisons. Partial Pearson correlation coefficients controlling for protein source (plant and animal) were used for the correlation analysis. Statistical significance was accepted at *P* < 0.05 for all procedures.

## Results

### Whole-body protein kinetics

**FIGURE 2:**
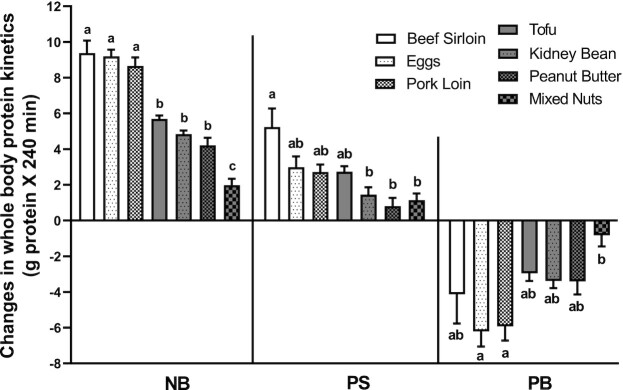
Changes in whole-body protein kinetics [net protein balance (NB), protein synthesis (PS), and protein breakdown (PB)] over postabsorptive values following protein meal consumption. One-factor repeated-measures ANOVA indicated a main effect of ounce-equivalent food source for NB, PS, and PB (*P* < 0.001). Groups not sharing the same letter are significantly different. Values (*n *= 8 per ounce-equivalent food source) are expressed as means ± SEMs.

Net whole-body protein balance increased above the baseline value after consumption of all the ounce-equivalent food sources (*P* < 0.001). Protein synthesis was stimulated by all protein food sources (*P* < 0.001), except for peanut butter, and protein breakdown was suppressed for all groups (*P* < 0.001) except for the mixed-nuts group. Overall, the anabolic response (i.e., increase in whole-body net protein balance) was greater in the groups that consumed animal proteins rather than plant proteins. NB increased more in the beef, pork, and eggs groups than all of the groups consuming plant-based protein food sources (*P* < 0.05). NB increased less in the mixed-nuts group than in all of the other groups. Protein synthesis increased more (*P* < 0.05) in the beef group than in the groups consuming kidney beans, peanut butter, or mixed nuts. Protein breakdown was suppressed more in those consuming eggs or pork loin than in the group consuming mixed nuts (*P* < 0.05) (**Figure 2**).

### Whole-body anabolic efficiency

**FIGURE 3:**
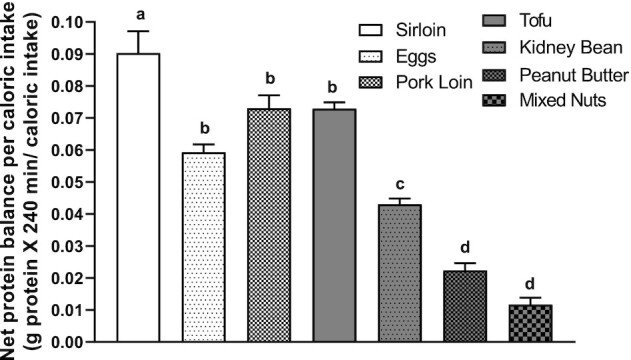
Whole-body net protein balance normalized by caloric intake. One-factor repeated-measures ANOVA indicated a main treatment effect in net balance normalized by caloric intake (*P* < 0.05). Groups not sharing the same letter are significantly different. Values (*n* = 8 per ounce-equivalent food source) are expressed as means ± SEMs.

Anabolic efficiency (net protein balance/caloric intake) was greater in the beef group than all the other groups (*P* < 0.05). The egg, pork loin, and tofu groups were greater for anabolic efficiency than the kidney bean, peanut butter, and mixed-nuts groups (*P* < 0.05). The kidney bean group was greater than the peanut butter and mixed-nuts groups (*P* < 0.05) (**Figure 3**).

### Plasma EAA and insulin responses

**FIGURE 4:**
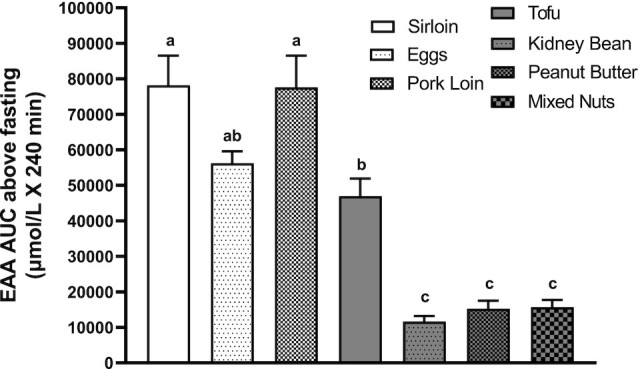
Plasma essential amino acid (EAA) temporal and AUC responses following protein meal intake. A factor-way ANOVA indicated a significant difference in the plasma EAA AUC response following ounce-equivalent food source ingestion (*P* < 0.01). Groups not sharing the same letter are significantly different. Values (*n* = 8 per ounce-equivalent food source) are expressed as means ± SEMs.

Plasma concentrations of EAAs, as well as the branched-chain amino acid (BCAA) and leucine, exhibited a significant main effect of ounce-equivalent food source (*P* < 0.01; **Figure 4** for EAA; **Supplemental Figure 2** for BCAA and leucine). All animal proteins and tofu induced higher EAA, BCAA, and leucine responses than the other plant proteins (*P* < 0.001). No statistical differences were observed in plasma insulin responses (**Supplemental Figure 3**).

### Correlations

**FIGURE 5:**
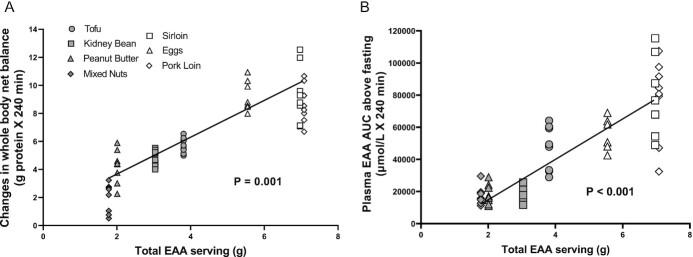
Relations between total essential amino acids (EAAs) per serving and the change in whole-body net balance (left panel) and between plasma EAA AUC over fasting value (right panel). Partial correlation coefficient analysis controlled for source (plant compared with animal) revealed significant associations between total EAA serving and changes in whole-body net balance (*r* = 0.44, *P* = 0.001) and plasma EAA AUC (*r* = 0.51. *P* < 0.001). *n*= 8 per ounce-equivalent food source.

A strong positive correlation was observed between EAA content in the ounce-equivalent food sources and the increase in net protein balance (*r* = 0.44, *P* = 0.001), as well as the plasma EAA AUC above baseline (*r* = 0.51, *P* < 0.001; **Figure 5**).

## Discussion

The main finding of this study is that the responses of whole-body net protein balance (anabolic response) to consumption of “ounce equivalents” of protein food sources as defined by the DGAs were not equivalent. Overall, animal-based protein food sources elicited greater anabolic responses than plant-based protein food sources. The greater anabolic responses were due to the simultaneous stimulation of whole-body protein synthesis and suppression of protein breakdown, which is consistent with our previous studies (10, 13–15). The magnitude of anabolic response was directly related to the EAA content of the protein food source, which was in turn reflected in the resulting plasma EAA concentrations (Figure 5 ). The relation between the anabolic response and plasma EAA concentrations is consistent with data demonstrating that the EAA component of protein is the driving stimulus for protein anabolism (13).

The findings of the current study are consistent with our previous work demonstrating that an egg-based breakfast induced a greater anabolic response than an isonitrogenous cereal-based breakfast, owing in great part to a higher peripheral EAA response (13). In the current study, the differences between each protein food source were minimized when the anabolic response was normalized to EAA content. This finding is consistent with other studies indicating that an equivalent amount of EAA intake, regardless of source, induces similar anabolic responses (16). In the current study, animal protein sources resulted in a greater whole-body anabolic response than plant protein sources because of greater EAA content.

The relation between the magnitude of anabolic response and the EAA content of the protein food sources suggests that metabolic equivalencies of different protein sources could reasonably be based on EAA content. However, to do so would result in large discrepancies in caloric values. The calories associated with each protein food source vary considerably (Table 2). The caloric values would vary even more so if equivalencies were based on EAA content. For example, if the EAA content of mixed nuts is normalized to the EAA content of 2 eggs, the caloric intake of mixed nuts would be 525 kcal, as opposed to 155 kcal in the case of the eggs. Since the major nutritional problem in the United States is obesity, it would be ill-advised to claim that 2 protein food sources were metabolically equivalent when the caloric value of one source is >3-fold greater than the other. The comparison of anabolic responses by means of whole-body protein kinetics was reasonable, since nutrients are consumed at the whole body level. However, our methodology did not include the amount of protein secreted into the gut in the process of digestion, and it did not include the amount of amino acids taken up directly from the gut by intestinal tissue and incorporated into protein before appearing in peripheral blood. We assumed these 2 processes were balanced. If the amount of direct uptake of amino acids and incorporation into protein actually exceeded the amount secreted (i.e., positive gut protein net balance), we underestimated the true anabolic response; however, this is unlikely because the measured gain in body protein (Figure 2) was ∼60% of the amount of ingested protein (Table 2) for all food sources, which represents greater nitrogen (N) retention than would have been expected on the basis of N-balance studies (17). Most important, our assumption regarding gut protein balance would be expected to have a similar impact on the calculated total anabolic response to all of the ounce-equivalent food sources.

There were potential limitations of our experimental design. We used young, healthy individuals as participants to be consistent with the experimental protocols used to define the RDA for protein (18). Although we have no reason to believe that the relations between net anabolic response and protein food source we observed are specific to that demographic, we cannot rule out that possibility. Also, we assessed the response to the ounce-equivalent protein sources as isolated foods, and they would normally be eaten in the context of a meal. Because the differing caloric values of the ounce equivalents had no discernable impact on our observed results, it is unlikely that the additional caloric intake of a complete meal would affect the importance of EAAs on the net anabolic response. On the other hand, it is possible that inclusion of additional protein food sources in a complete meal could provide a sufficient amount of the limiting amino acid of the ounce-equivalent protein food source to enhance the anabolic response. For example, it is possible that the addition of an animal-based protein would enhance the anabolic impact of a plant-based protein, because lysine is often the limiting amino acid of a plant-based protein and is rarely limiting in animal-based proteins. The DGAs do not discuss the issue of the potential interactions of complementary proteins, and this topic is beyond the scope of our article. Finally, we have assessed the acute anabolic response to a single meal. We have previously shown that the acute determination of protein kinetics in response to consumption of free EAAs translates to long-term functional outcomes (19), but long-term outcomes of consumption of different ounce-equivalent protein food sources remain to be determined.

There is no basis for believing that any of the potential limitations in tracer methodology or experimental design discussed above affected our fundamental conclusion that the “ounce equivalents” as promulgated by the DGAs are not metabolically equivalent in terms of either the anabolic response or caloric value. Thus, the current labeling of the “ounce-equivalent” protein food sources in the DGAs is misleading to the consumer and the practitioner and should be modified. However, the most effective way to present protein food sources in terms of more realistic “metabolic equivalencies” is unclear. Presenting a quick and easy comparator of protein food sources is useful to the average consumer, but the information presented should be accurate. A more informative approach than the current DGAs may be to discuss protein food sources in terms of “high quality” and “low quality,” as determined by the DIAAS (5). Alternatively, the magnitude of variations in EAA content and caloric value of protein food sources may make the assignment of metabolic equivalencies fundamentally unrealistic. In any case, the importance of distinguishing between high- and low-quality proteins should be included as the DGAs evolve toward tools designed to establish healthy eating patterns.

## Conclusion

We conclude that “ounce equivalents” of protein food sources as expressed in the DGAs are not metabolically equivalent in young, healthy participants. The magnitude of anabolic response to specific dietary proteins should be considered in the development of new DGAs with regard to recommended Healthy Eating Patterns, particularly those for which protein sources are plant based.

## Supplementary Material

nxaa401_Supplemental_Files

## Notes

Funding: Supported by the National Pork Board (AAF), Egg Nutrition Center (AAF), and Beef Checkoff (National Cattlemen's Beef Association) (RRW).

Author disclosures: RRW, received travel expenses and honorarium from the National Cattlemen's Beef Association for giving a talk and is a shareholder in Essential Blends, LLC and The Amino Company, LLC. All other authors report no conflicts of interest.

Supplemental Figures 1–3 are available from the “Supplementary data” link in the online posting of the article and from the same link in the online table of contents at https://academic.oup.com/ajcn/.

Abbreviations used: BCAA, branched-chain amino acid; DGAs, Dietary Guidelines for Americans; DIAAS, digestible indispensable amino acid score; EAA, essential amino acid; NB, net balance; PDCAAS, protein digestibility–corrected amino acid score; Phe, phenylalanine; Ra, rate of appearance; RIOA, Donald W. Reynolds Institute on Aging; SPE, splanchnic extraction; UAMS, University of Arkansas for Medical Sciences.
