# Supplementary figures and images for: Metabolic Evaluation of the Dietary Guidelines’ Ounce Equivalents of Protein Food Sources in Young Adults: A Randomized Controlled Trial
Source: J Nutr. 2021 Mar 9;151(5):1190–6. doi: 10.1093/jn/nxaa401 (PMC8112772; doi:10.1093/jn/nxaa401)

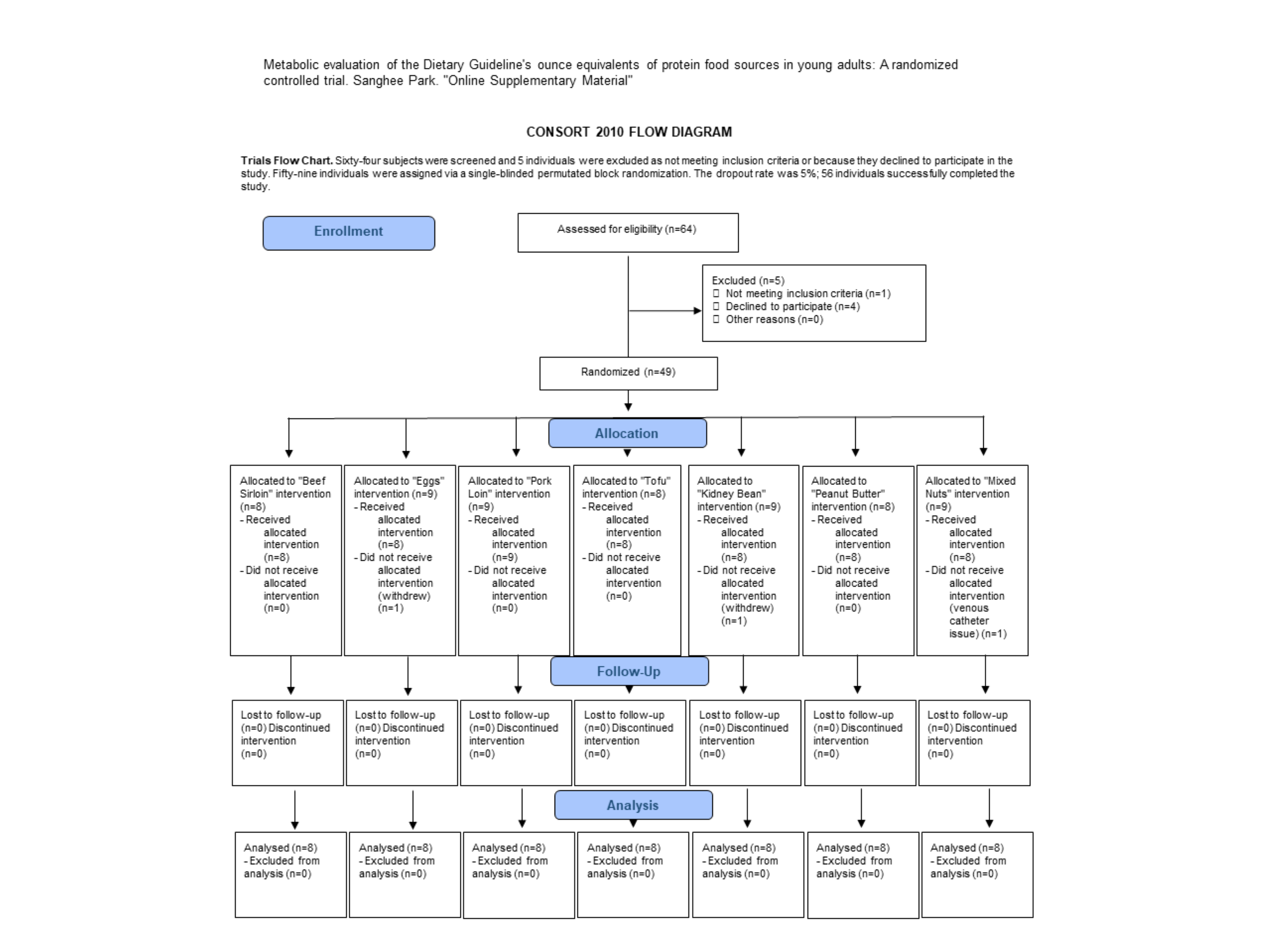

Supplement: nxaa401_Supplemental_Files [file nxaa401_supplemental_files.zip › Supplemental Figure 1 vR3 p2400.tif]

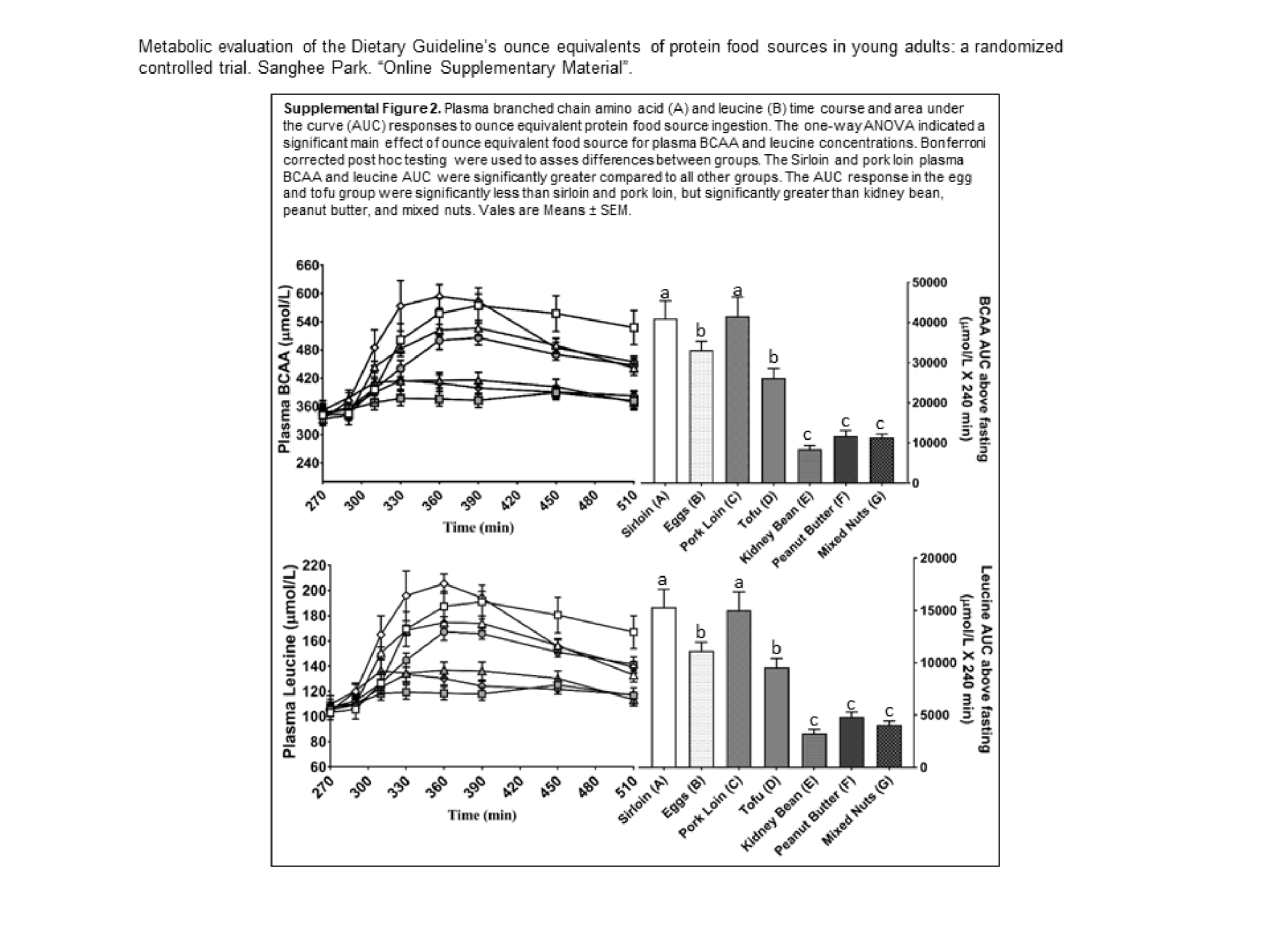

Supplement: nxaa401_Supplemental_Files [file nxaa401_supplemental_files.zip › Supplemental Figure 2 vR3 p2400.tif]

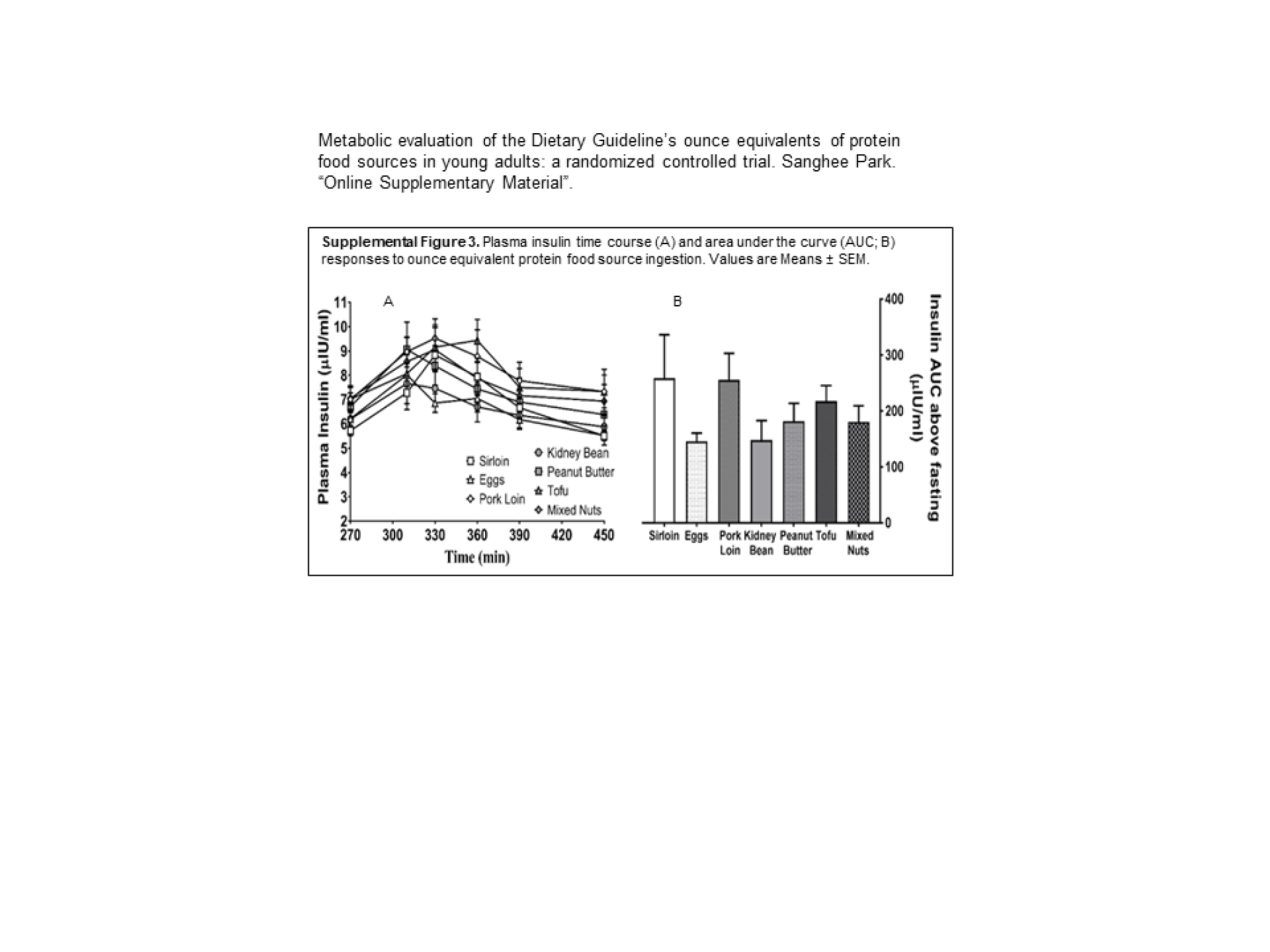

Supplement: nxaa401_Supplemental_Files [file nxaa401_supplemental_files.zip › Supplemental Figure 3 vR3 p2400.tif]
